# $\ell _1$-regularized recursive total least squares based sparse system identification for the error-in-variables

**DOI:** 10.1186/s40064-016-3120-6

**Published:** 2016-08-31

**Authors:** Jun-seok Lim, Hee-Suk Pang

**Affiliations:** Department of Electronics Engineering, Sejong University, Kunja, Kwangjin, 98, 143-747 Seoul, Korea

**Keywords:** Adaptive filter, TLS, RLS, Convex regularization, Sparsity, $\ell$1-norm

## Abstract

In this paper an $\ell _1$-regularized recursive total least squares (RTLS) algorithm is considered for the sparse system identification. Although recursive least squares (RLS) has been successfully applied in sparse system identification, the estimation performance in RLS based algorithms becomes worse, when both input and output are contaminated by noise (the error-in-variables problem). We proposed an algorithm to handle the error-in-variables problem. The proposed $\ell _1$-RTLS algorithm is an RLS like iteration using the $\ell _1$ regularization. The proposed algorithm not only gives excellent performance but also reduces the required complexity through the effective inversion matrix handling. Simulations demonstrate the superiority of the proposed $\ell _1$-regularized RTLS for the sparse system identification setting.

## Background

There has been a recent interest in adaptive algorithms to handle sparsity in various signals and systems (Gu et al. 2009; Chen et al. 2009; Babadi et al. 2010; Angelosante et al. 2010; Eksioglu 2011; Eksioglu and Tanc 2011; Kalouptsidis et al. 2011). The idea is to exploit a priori knowledge about sparsity in a signal that needs to be processed for system identification. Several algorithms based on the least-mean square (LMS) (Gu et al. 2009; Chen et al. 2009) and the recursive least squares (RLS) (Babadi et al. 2010; Angelosante et al. 2010; Eksioglu 2011; Eksioglu and Tanc 2011) techniques have been reported with different penalty or shrinkage functions. In a broad range of signal processing applications, not only the system output is corrupted by measurement noise, but also the measured input signal may often be corrupted by the additive noise due to such as sampling error, quantization error and wide-band channel noise. However, the algorithms for sparsity can handle only the corrupted output case. It is necessary to derive an algorithm to handle a noisy case of both noisy input and noisy output (i.e. the error-in-variables problem).

One of the potential counterparts to handle the error-in-variables problem is the total-least-squares estimator (TLS) that seeks to minimize the sum of squares of residuals on all of the variables in the equation instead of minimizing the sum of squares of residuals on only the response variable. Golub and Van Loan introduced the TLS problem to the field of numerical analysis (Golub and Loan 1980); consequently, other researchers have developed and analyzed adaptive algorithms that employ the TLS formulation and its extensions (Dunne and Williamson 2000, 2004; Feng et al. 1998; Arablouei et al. 2014; Davila 1994; Arablouei et al. 2015). Recursive based algorithms were also studied along with adaptive TLS estimators (Soijer 2004; Choi et al. 2005). The algorithms were denoted as recursive total least squares (RTLS) or sequential total least squares (STLS). These algorithms recursively calculate and track the eigenvector corresponding to the minimum eigenvalue (the TLS solution) from the inverse covariance matrix of the augmented sample matrix. Some TLS based algorithms were proposed for the sparse signal processing (Tanc 2015; Arablouei 2016; Zhu et al. 2011; Dumitrescu 2013; Lim and Pang 2016). The algorithms in Tanc (2015), Arablouei (2016) utilized the gradient based method. The algorithms in Zhu et al. (2011), Dumitrescu (2013) were based on the block coordinate descent method. In Lim and Pang (2016), the TLS method was applied to handle the group sparsity problem.

In this paper, we consider the $\ell _1$ regularization for the RTLS cost function, in which the recursive procedure is derived from the generalized eigendecomposition method in Davila (1994) and Choi et al. (2005), and the regularization approach outlined in Eksioglu and Tanc (2011) is used in order to handle the sparsity. We develop the update algorithm for the $\ell _1$-regularized RTLS using results from subgradient calculus. As a result, we propose the algorithm superior to the algorithm of Eksioglu and Tanc (2011) in the error-in-variables. We also reduce the total complexity by utilizing the inverse matrix update effectively. The proposed algorithm improves the sparse system estimation performance in the error-in-variables with only a little additional complexity. We provide simulation results to examine the performance of the proposed algorithm in comparison with the algorithm of Eksioglu and Tanc (2011).

## Sparse system identification problem

In the sparse system identification problem of interest, the system observes a signal represented by an $M\times 1$ vector ${\mathbf{x}}(k) = [x_1 (k), \ldots ,x_M (k)]^T$ at time instant *k*, performs filtering and obtains the output $y(k) = {\mathbf{x}}^T(k){\mathbf{w}}_o (k)$, where ${\mathbf{w}}_o (k) = [w_1 (k), \ldots ,w_M (k)]^T$ is an M–length finite-impulse-response (FIR) system that represents the actual system. For system identification, an adaptive filter with M coefficients ${\hat{\mathbf{w}}}(k)$ is employed in such a way that observes ${\mathbf{x}}(k)$ and produces an estimate $\hat{y}(k) = {\mathbf{x}}^T(k){{\hat{\mathbf{w}}}}(k)$. The system identification scheme then compares the output of the actual system *y*(*k*) and the adaptive filter $\hat{y}(k)$, resulting in an error signal $e(k) = y(k) + n(k) - \hat{y}(k) = \tilde{y}(k) - \hat{y}(k)$, where *n*(*k*) is the measurement noise. In this context, the goal of an adaptive algorithm is to identify the system by minimizing the cost function defined by

1 $$\begin{aligned} {{\hat{\mathbf{w}}}} = \arg \mathop {\min }\limits _{{\hat{\mathbf{w}}}} \frac{1}{2}\sum \limits _{m = 0}^k {\lambda ^{k - m}\left( {e\left( m \right) } \right) ^2}. \end{aligned}$$

The gradient based minimization derives the following equation.

2 $$\begin{aligned} {{{\varvec{\Phi }}}}\left( k \right) {{\hat{\mathbf{w}}}}\left( k \right) = {\mathbf{r}}(k), \end{aligned}$$

where ${{{\varvec{\Phi }} }}\left( k \right) = \sum \nolimits _{m = 0}^k {\lambda ^{k - m}{\mathbf{x}}(m){\mathbf{x}}^T(m)}$ and ${\mathbf{r}}(k) = \sum \nolimits _{m = 0}^k {\lambda ^{k - m}\tilde{y}(m){\mathbf{x}}(m)}$. This equation is the matrix form of the normal equations for least squares solution.

Especially, we call *M*-th order ${\mathbf{w}}_o (k)$ sparse system, when the number of nonzero coefficients $K \ll M$. In order to estimate the *M*-th order sparse system, most estimation algorithms exploit non-zero coefficients of the system to obtain performance benefits and/or a computational complexity reduction (Gu et al. 2009; Chen et al. 2009; Babadi et al. 2010; Angelosante et al. 2010; Eksioglu 2011; Eksioglu and Tanc 2011).

## $\ell _1$-regularized RTLS (recursive total least squares)

**Fig. 1:**
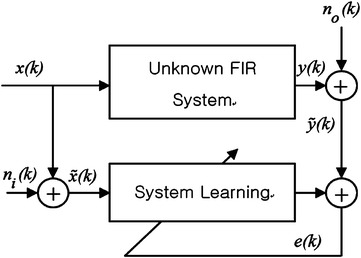
The model of noisy input and noisy output system

In TLS, we assume a given unknown system that both the input and output are corrupted by noise. The system should be estimated from the noisy observation of the input and the output (Fig. 1). In this system output is given by:

3 $$\begin{aligned} \tilde{y}(k) = {{\tilde{\mathbf{x}}}}^T(k){\mathbf{w}}_o + n_o (k), \end{aligned}$$

where the output noise $n_{o}(k)$ is the Gaussian white noise with variance $\sigma _o^2$ and independent of the input signal. The noisy input of the system is given by:

4 $$\begin{aligned} {\tilde{x}}(k) = {x}(k) + {n}_i (k) , \end{aligned}$$

where the input noise $n_{i}(k)$ is the Gaussian white noise with variance $\sigma _i^2$.

For TLS solution, the augmented data vector is considered as:

5 $$\begin{aligned} \overline{\mathbf{x}} (k) = \left[ {{{\tilde{\mathbf{x}}}}^T(k),\tilde{y}(k)} \right] ^T \,\in\, \text{ R }^{(M + 1)\times 1}. \end{aligned}$$

And its covariance matrix has the following structure:

6 $$\begin{aligned} \overline{\mathbf{R}} = \left[ {{\begin{array}{*{20}c} {{\tilde{\mathbf{R}}}} & {\mathbf{p}} \\ {{\mathbf{p}}^T} & c \\ \end{array} }} \right] , \end{aligned}$$

where ${\mathbf{p}} = E\left\{ {{{\tilde{\mathbf{x}}}}(k)y(k)} \right\}$ and $c = E\left\{ {y(k)y(k)} \right\}$, ${{\tilde{\mathbf{R}}}} = E\left\{ {{{\tilde{\mathbf{x}}}}(k){{\tilde{\mathbf{x}}}}^T(k)} \right\} = {\mathbf{R}} + \sigma _i^2 {\mathbf{I}}$, ${\mathbf{R}} = E\left\{ {{\mathbf{x}}(k){\mathbf{x}}^T(k)} \right\}$. In Davila (1994) and Choi et al. (2005), the TLS problem is thus reduced to finding the eigenvector that is associated with the smallest eigenvalue of ${{\bar{\mathbf{R}}}}$. The following equation is the simplified cost function to find the eigenvector that is associated with the smallest eigenvalue of ${{\bar{\mathbf{R}}}}$.

7 $$\begin{aligned} \tilde{J}({\mathbf{w}}) = \frac{{{\tilde{\mathbf{w}}}}^T\overline{\mathbf{R}} {{\tilde{\mathbf{w}}}}}{{{\tilde{\mathbf{w}}}}^T\overline{\mathbf{D}} {{\tilde{\mathbf{w}}}}} = \frac{[{\mathbf{w}}^T, - 1]\overline{\mathbf{R}} [{\mathbf{w}}^T, - 1]^T}{[{\mathbf{w}}^T, - 1]\overline{\mathbf{D}} [{\mathbf{w}}^T, - 1]^T}, \end{aligned}$$

where $$\overline{\mathbf{D}} = \left[ {{\begin{array}{*{20}c} {\mathbf{I}} & {\mathbf{0}} \\ {\mathbf{0}} & \gamma \\ \end{array} }} \right]$$ with $\gamma = \frac{\sigma _o^2 }{\sigma _i^2 }$. Minimum value of (7) is recognized as the smallest generalized eigenvalue of $\overline{\mathbf{R}}$ (Dunne and Williamson 2004). Therefore, we can find the eigenvector associated with the smallest eigenvalue. The smallest eigenvector can also be derived from the maximization of (8).

8 $$\begin{aligned} \tilde{J}({\mathbf{w}}) = \frac{{{\tilde{\mathbf{w}}}}^H\overline{\mathbf{D}} {{\tilde{\mathbf{w}}}}}{{{\tilde{\mathbf{w}}}}^H\overline{\mathbf{R}} {{\tilde{\mathbf{w}}}}} = \frac{[{\mathbf{w}}^H, - 1]\overline{\mathbf{D}} [{\mathbf{w}}^T, - 1]^T}{[{\mathbf{w}}^H, - 1]\overline{\mathbf{R}} [{\mathbf{w}}^T, - 1]^T}. \end{aligned}$$

We adopt the modified cost function by the addition of a penalty function. This penalty function can be chosen to reflect a priori knowledge about the true sparsity system.

9 $$\begin{aligned} \tilde{J}({\mathbf{w}}) = \frac{[{\mathbf{w}}^H, - 1]\overline{\mathbf{D}} [{\mathbf{w}}^T, - 1]^T}{[{\mathbf{w}}^H, - 1]\overline{\mathbf{R}} [{\mathbf{w}}^T, - 1]^T} + \gamma f\left( {{\tilde{\mathbf{w}}}} \right) = \frac{{{\tilde{\mathbf{w}}}}^H\overline{\mathbf{D}} {{\tilde{\mathbf{w}}}}}{{{\tilde{\mathbf{w}}}}^H\overline{\mathbf{R}} {{\tilde{\mathbf{w}}}}} + \gamma f\left( {{\tilde{\mathbf{w}}}} \right) , \end{aligned}$$

where $\gamma$ is the regularized parameter in Eksioglu and Tanc (2011). We adopt the $\ell _1$ penalty function as follows:

10 $$\begin{aligned} f({{\tilde{\mathbf{w}}}}) = \left\| {{\tilde{\mathbf{w}}}} \right\| _1 = \sum \limits _{i = 0}^{M - 1} {\left| {\tilde{w}_i } \right| }. \end{aligned}$$

We solve the equations by $\nabla _{{\tilde{\mathbf{w}}}} J(k) = 0$,

11 $$\begin{aligned} \nabla _{{\tilde{\mathbf{w}}}} J(k)& = 0: \frac{\overline{\mathbf{D}} {{\tilde{\mathbf{w}}}}(k)\left( {{{\tilde{\mathbf{w}}}}^H(k)\overline{\mathbf{R}} (k){{\tilde{\mathbf{w}}}}(k)} \right) }{\left( {{{\tilde{\mathbf{w}}}}^H(k)\overline{\mathbf{R}} (k){{\tilde{\mathbf{w}}}}(k)} \right) ^2} - \frac{{{\tilde{\mathbf{w}}}}^H(k)\overline{\mathbf{D}} {{\tilde{\mathbf{w}}}}(k)\left( {\overline{\mathbf{R}} (k){{\tilde{\mathbf{w}}}}(k)} \right) }{\left( {{{\tilde{\mathbf{w}}}}^H(k)\overline{\mathbf{R}} (k){{\tilde{\mathbf{w}}}}(k)} \right) ^2} \nonumber \\& \quad + \gamma _k \nabla f\left( {{{\tilde{\mathbf{w}}}}(k)} \right) = 0. \end{aligned}$$

where ${{\bar{\mathbf{R}}}}(k) = \lambda {{\bar{\mathbf{R}}}}(k - 1) + {{\bar{\mathbf{x}}}}(k){{\bar{\mathbf{x}}}}^H(k)$, ${{\tilde{\mathbf{w}}}}(k) = \left[ {{{\hat{\mathbf{w}}}}^T(k), - 1} \right] ^T$and ${{\hat{\mathbf{w}}}}(k)$ is the estimation result for the unknown system at *k*-th time step. The subgradient of $f({{\tilde{\mathbf{w}}}})$ is $\nabla ^s\left\| {{\tilde{\mathbf{w}}}} \right\| _1 = \mathrm{sgn}({{\tilde{\mathbf{w}}}})$ (Babadi et al. 2010; Kalouptsidis et al. 2011), and sgn ($\cdot$) is the component-wise sign function. In (11), the regularized parameter,$\gamma _k$, is time-varying, which governs a tradeoff between the approximation error and the penalty function. From (11), we obtain

12 $$\begin{aligned} {{\tilde{\mathbf{w}}}}(k)& = \frac{{{\tilde{\mathbf{w}}}}^H(k)\overline{\mathbf{R}} (k){{\tilde{\mathbf{w}}}}(k)}{\left\| {{{\tilde{\mathbf{w}}}}(k)} \right\| ^2}\left( {\overline{\mathbf{R}} ^{ - 1}(k){{\tilde{\mathbf{w}}}}(k)} \right) \nonumber \\& \quad + \gamma _k \frac{\left( {{{\tilde{\mathbf{w}}}}^H(k)\overline{\mathbf{R}} (k){{\tilde{\mathbf{w}}}}(k)} \right) ^2}{\left\| {{{\tilde{\mathbf{w}}}}(k)} \right\| ^2}\overline{\mathbf{R}} ^{ - 1}(k)\nabla f\left( {{{\tilde{\mathbf{w}}}}(k)} \right) . \end{aligned}$$

And we obtain the estimated parameter of the unknown system as

13 $$\begin{aligned} {{\hat{\mathbf{w}}}}(k) = - {{\tilde{\mathbf{w}}}}_{1:M} (k) / \tilde{w}_{M + 1} (k). \end{aligned}$$

As the estimated unknown system can be derived from the ratio between $\tilde{w}_{M + 1} (k)$ and the elements in ${{\tilde{\mathbf{w}}}}_{1:M} (k)$, the normalization of ${{\tilde{\mathbf{w}}}}(k)$ as ${{\tilde{\mathbf{w}}}}(k) = {{\tilde{\mathbf{w}}}}(k) / \left\| {{{\tilde{\mathbf{w}}}}(k)} \right\|$ keeps the same solution in (13) as well as numerical stability in the iteration. By applying the normalization of (12) becomes,

14 $$\begin{aligned} {{\tilde{\mathbf{w}}}}(k)= & \left( {{{\tilde{\mathbf{w}}}}^H(k)\overline{\mathbf{R}} (k){{\tilde{\mathbf{w}}}}(k)} \right) \times \nonumber \\&\left( {\overline{\mathbf{R}} ^{ - 1}(k){{\tilde{\mathbf{w}}}}(k) + \gamma _k \left( {{{\tilde{\mathbf{w}}}}^H(k)\overline{\mathbf{R}} (k){{\tilde{\mathbf{w}}}}(k)} \right) \overline{\mathbf{R}} ^{ - 1}(k)\nabla f\left( {{{\tilde{\mathbf{w}}}}(k)} \right) } \right) . \end{aligned}$$

In addition, we can approximate (14) as follows.

15 $$\begin{aligned} {{\tilde{\mathbf{w}}}}(k)&\approx \left( {\overline{\mathbf{R}} ^{ - 1}(k){{\tilde{\mathbf{w}}}}(k - 1)} \right) \nonumber \\&\quad + \gamma _k \left( {{{\tilde{\mathbf{w}}}}^H(k - 1)\overline{\mathbf{R}} (k - 1){{\tilde{\mathbf{w}}}}(k - 1)} \right) \overline{\mathbf{R}} ^{ - 1}(k)\nabla f\left( {{{\tilde{\mathbf{w}}}}(k - 1)} \right) , \end{aligned}$$

where $${{\bar{\mathbf{R}}}}(k) = \lambda {{\bar{\mathbf{R}}}}(k - 1) + {{\bar{\mathbf{x}}}}(k){{\bar{\mathbf{x}}}}^H(k)$$. In (15),

16 $$\begin{aligned} \begin{aligned} {{\tilde{\mathbf{w}}}}^H(k){{\bar{\mathbf{R}}}}(k){{\tilde{\mathbf{w}}}}(k) & =\lambda {{\tilde{\mathbf{w}}}}^H(k){{\bar{\mathbf{R}}}}(k - 1){{\tilde{\mathbf{w}}}}(k) + {{\tilde{\mathbf{w}}}}^H(k){{\bar{\mathbf{x}}}}(k){{\bar{\mathbf{x}}}}^H(k){{\tilde{\mathbf{w}}}}(k) \\ &\approx \lambda {{\tilde{\mathbf{w}}}}^H(k - 1){{\bar{\mathbf{R}}}}(k - 1){{\tilde{\mathbf{w}}}}(k - 1) + {{\tilde{\mathbf{w}}}}^H(k){{\bar{\mathbf{x}}}}(k){{\bar{\mathbf{x}}}}^H(k){{\tilde{\mathbf{w}}}}(k) \\ & =\lambda {{\tilde{\mathbf{w}}}}^H(k - 1){{\bar{\mathbf{R}}}}(k - 1){{\tilde{\mathbf{w}}}}(k - 1) + \bar{y}(k)^2.\\ \end{aligned} \end{aligned}$$

In (11), we apply the same $\gamma _k$ in Eksioglu and Tanc (2011).

17 $$\begin{aligned} \gamma _k = \frac{2\frac{tr\left( {{{\bar{\mathbf{R}}}}^{ - 1}(k)} \right) }{M}\left( {f\left( {{{\hat{\mathbf{w}}}}_{aug} (k)} \right) - \rho } \right) + \nabla ^sf\left( {{{\hat{\mathbf{w}}}}_{aug} (k)} \right) {{\bar{\mathbf{R}}}}^{ - 1}(k){{\varepsilon }}(k)}{\left\| {{{\bar{\mathbf{R}}}}^{ - 1}(k)\nabla ^sf\left( {{{\hat{\mathbf{w}}}}_{aug} (k)} \right) } \right\| _2^2 }, \end{aligned}$$

where ${{\hat{\mathbf{w}}}}_{aug} (k) = \left[ {{{\hat{\mathbf{w}}}}^T(k), - 1} \right] ^T$, ${{\hat{\mathbf{w}}}}_{aug,RLS} (k) = \left[ {{{\hat{\mathbf{w}}}}_{RLS}^T (k), - 1} \right] ^T$, ${{\varepsilon }}(k) = {{\hat{\mathbf{w}}}}_{aug} (k) - {{\hat{\mathbf{w}}}}_{aug,RLS} (k)$, and ${{\hat{\mathbf{w}}}}_{RLS} (k)$ is the parameter estimated by recursive least squares (RLS).

## A simplified way to solve $\ell _1$-regularized RTLS

The proposed algorithm needs a solution in (15) as well as RLS solution for ${{\hat{\mathbf{w}}}}_{RLS} (k)$ in $\gamma _k$. However, this makes the algorithm complex and we find a less complex way from block matrix inversion lemma in Moon and Stirling (2000). The required calculation complexity can be simplified if we use the following matrix manipulation: ${{\bar{\mathbf{X}}}}(k) = \left[ {{{\tilde{\mathbf{X}}}}(k)^T \vdots {{\tilde{\mathbf{y}}}}(k)} \right] ^T$ and $${{\bar{\mathbf{X}}}}(k){{\bar{\mathbf{X}}}}^T(k) = \left[ {{\begin{array}{*{20}c} {{{\tilde{\mathbf{X}}}}(k){{\tilde{\mathbf{X}}}}^T(k)} & {{{\tilde{\mathbf{X}}}}(k){{\tilde{\mathbf{y}}}}(k)} \\ {{{\tilde{\mathbf{y}}}}^T(k){{\tilde{\mathbf{X}}}}^T(k)} & {{{\tilde{\mathbf{y}}}}^T(k){{\tilde{\mathbf{y}}}}(k)} \\ \end{array} }} \right] = \left[ {{\begin{array}{*{20}c} {{{\tilde{\mathbf{X}}}}(k){{\tilde{\mathbf{X}}}}^T(k)} & {\mathbf{a}} \\ {{\mathbf{a}}^T} & c \\ \end{array} }} \right]$$, then:

18 $$\begin{aligned} {{\bar{\mathbf{R}}}}^{ - 1}(k)& = \left( {{{\bar{\mathbf{X}}}}(k){{\bar{\mathbf{X}}}}(k)^T} \right) ^{ - 1} \nonumber \\& = \left[ {{\begin{array}{*{20}l} {{\mathbf{P}}_{xx} (k) + \beta {\mathbf{P}}_{xx} (k){\mathbf{aa}}^T{\mathbf{P}}_{xx} (k)} & {-\beta {\mathbf{P}}_{xx} (k){\mathbf{a}}}\nonumber \\ { - \beta {\mathbf{a}}^T{\mathbf{P}}_{xx} (k)} & \beta \\ \end{array} }} \right] \nonumber \\& = \left[ {{\begin{array}{*{20}c} {{\mathbf{A}}_{11} } & {{\mathbf{A}}_{12} } \\ {{\mathbf{A}}_{21} } & {{\mathbf{A}}_{22} } \\ \end{array} }} \right] , \end{aligned}$$

where ${{\tilde{\mathbf{X}}}}(k) = \left[ {{{\tilde{\mathbf{x}}}}(k),\sqrt{\lambda }{{\tilde{\mathbf{x}}}}(k - 1), \cdots ,\left( {\sqrt{\lambda }} \right) ^k{{\tilde{\mathbf{x}}}}(0)} \right]$, $\beta = \left( {c - {\mathbf{a}}^H\left( {{{\tilde{\mathbf{X}}}}(k){{\tilde{\mathbf{X}}}}^T(k)} \right) ^{ - 1}{\mathbf{a}}} \right) ^{ - 1}$. ${\mathbf{A}}_{12} = - \beta \left( {{{\tilde{\mathbf{X}}}}(k){{\tilde{\mathbf{X}}}}(k)^T} \right) ^{ - 1}{\mathbf{a}}$ in (18) includes

19 $$\begin{aligned} \left( {{{\tilde{\mathbf{X}}}}(k){{\tilde{\mathbf{X}}}}(k)^T} \right) ^{ - 1}{\mathbf{a}} = \left( {{{\tilde{\mathbf{X}}}}(k){{\tilde{\mathbf{X}}}}(k)^T} \right) ^{ - 1}{{\tilde{\mathbf{X}}}}(k){{\tilde{\mathbf{y}}}}(k), \end{aligned}$$

from which the RLS solution, ${{\hat{\mathbf{w}}}}_{RLS} (k)$ can be derived by dividing ${\mathbf{A}}_{12}$with the constant $- \beta$. When we solve the proposed $\ell _1$-RTLS in (15–17), $\gamma _k$ in (17) needs to solve the RLS for ${{\varepsilon }}(k)$. Therefore, all procedures in (15–17) need $2M^2 + 2M$ additional multiplications. However, the simplification in this section needs only an additional division.

## Simulation results

In this experiment, we follow the experiment scenario in Eksioglu and Tanc (2011). The true system function ${\mathbf{w}}$ has a total of N = 64 taps, where only S of them are nonzero. The nonzero coefficients are positioned randomly and take their values from an $N(0,1/\mathrm{S})$ distribution. The input signal is $x_k \sim N(0,1)$. Noise is added to both the input and the output, and the additive input and output noises in this paper are $n_{in,k} \sim N(0,\sigma _{in}^2 )$ and $n_{out,k} \sim N(0,\sigma _{out}^2 )$, respectively. These additional noises are necessary to experiment the errors-in-variables problem. The proposed $\ell _1$-RTLS algorithm is realized with the automatic $\gamma _k$ using (17). The $\rho$ value in (17) is taken to be the true value of $f({\mathbf{w}})$ as in Eksioglu and Tanc (2011), that is $\rho = \left\| {{\mathbf{w}}_o } \right\| _1$ for the $\ell _1$-RTLS. We also compare the ordinary RLS and the $\ell _1$-RLS of Eksioglu and Tanc (2011) with the proposed $\ell _1$-RTLS.

**Fig. 2:**
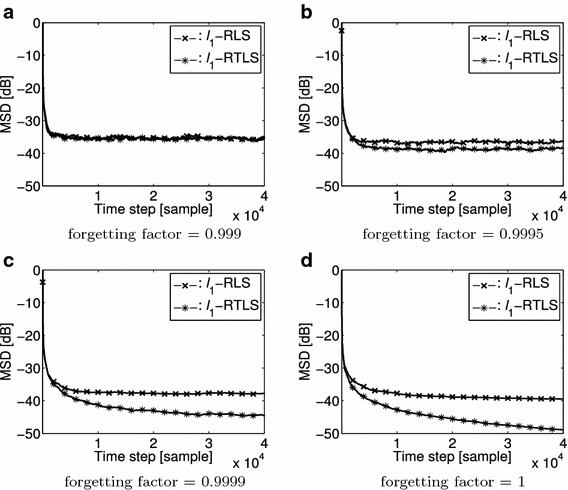
Steady-State MSD for S =2 with different forgetting factors. **a** forgetting factor = 0.999. **b** forgetting factor = 0.9995. **c** forgetting factor = 0.9999. **d** forgetting factor = 1

The proposed algorithm needs the inversion of the covariance matrix as (15) in order to derive the eigenvector. The better covariance matrix is needed for the better eigenvector. Therefore, the forgetting factor is needed close to 1. Figure 2 compares the estimation performance results between the $\ell _1$-RLS and the $\ell _1$-RTLS in mean square deviation (MSD) with S = 4 and the different forgetting factors. We add the noise at both input and output with $\sigma _{in} = \sigma _{out} = 0.1$. For this comparison, we set the forgetting factor to 0.999, 0.9995, 0.9999 and 1, respectively. Figure 2 shows that the performance becomes better as the forgetting factor goes to 1 although the proposed $\ell _1$-RTLS becomes better when the forgetting factor is greater than 0.999.

**Fig. 3:**
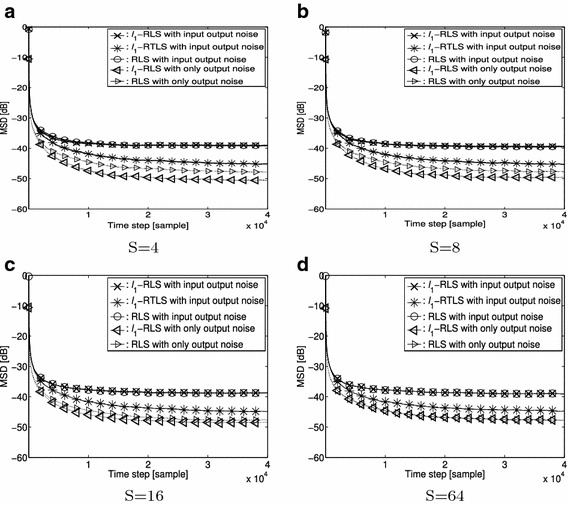
Steady-State MSD for S = 4, 8, 16 and 64 with forgetting factor of 0.9999. **a** S = 4. **b** S = 8. **c** S = 16. **d** S = 64

In Fig. 3 we simulate the algorithms with $\sigma _{in} = \sigma _{out} = 0.1$ and for S = 4, 8, 16 and 64, where S = 64 corresponds to a completely non-sparse system. In this simulation we set the forgetting factor to 0.9999. Figure 3a–d plot the MSD curves of the proposed $\ell _1$-RTLS, the $\ell _1$-RLS and the ordinary RLS with the different S values. Figure 3 includes the MSD curves from the $\ell _1$-RLS and the ordinary RLS with the contaminated output only, and shows the estimation performance of the $\ell _1$-RLS and the ordinary RLS significantly deteriorates when input and output are contaminated with noise. The proposed $\ell _1$-RTLS, however, outperforms the $\ell _1$-RLS and the ordinary RLS when input and output are contaminated with noise.

Table 1 summarizes the steady-state MSD values at the end of 500 independent trials for the algorithms. In this simulation, we set the forgetting factor to 0.999, 0.9995, 0.9999 and 1 and vary the sparsity S to 4, 8, 16 and 64, respectively. Table 1 shows the performance of the $\ell _1$-RLS is almost the same as that of the ordinary RLS. This means the $\ell _1$-RLS cannot improve the estimation performance when both input and output are contaminated by noise. However, the proposed $\ell _1$-RTLS outperforms the other algorithms. The improvement goes better as the forgetting factor gets close to 1.

**Table 1:** Comparison of MSD in case of noisy input and noisy output

Forgetting factor	Algorithm	S = 4 (dB)	S = 8 (dB)	S = 16 (dB)	S = 64 (dB)
0.999	RLS	−34.0	−33.5	−33.8	−33.7
	$\ell _1$-RLS	−34.8	−33.6	−33.5	−33.7
	$\ell _1$-RTLS	−35.4	−35.0	−34.9	−34.8
	($\ell _1$-RLS)-($\ell _1$-RTLS)	0.6	1.4	1.4	1.1
0.9995	RLS	−35.7	−35.9	−36.1	−35.9
	$\ell _1$-RLS	−36.0	−35.8	−35.8	−35.9
	$\ell _1$-RTLS	−38.1	−38.0	−38.0	−38.0
	($\ell _1$-RLS)-($\ell _1$-RTLS)	2.1	2.2	2.2	2.1
0.9999	RLS	−39.0	−39.3	−38.6	−38.6
	$\ell _1$-RLS	−38.9	−39.1	−38.5	−38.6
	$\ell _1$-RTLS	−44.0	−44.1	−43.6	−43.6
	($\ell _1$-RLS)-($\ell _1$-RTLS)	5.1	5.0	5.1	5.0
1	RLS	−39.0	−38.5	−38.6	−38.9
	$\ell _1$-RLS	−39.0	−38.5	−38.6	−38.9
	$\ell _1$-RTLS	−45.0	−44.6	−44.8	−44.7
	($\ell _1$-RLS)-($\ell _1$-RTLS)	6.0	6.1	6.2	5.8

*$\ell _1$-RLS is the algorithm in Eksioglu and Tanc (2011)

## Conclusion

In this paper, we propose an $\ell _1$-regularized RTLS for sparse system identification. The proposed algorithm keeps good performance in case of both noisy input and noisy output. We develop the recursive procedure for total least squares solution with an $\ell _1$-regularized cost function. We also present a simplified solution requiring only a little additional complexity in order to integrate the regularization factor. Simulations show that the introduced $\ell _1$-regularized RTLS algorithm shows better performance than RLS and $\ell _1$-regularized RLS in the sparse system with noisy input and noisy output.
